# Fatty Acid Synthase Mutations Predict Favorable Immune Checkpoint Inhibitor Outcome and Response in Melanoma and Non-Small Cell Lung Cancer Patients

**DOI:** 10.3390/cancers14225638

**Published:** 2022-11-17

**Authors:** Qinghua Wang, Na Tian, Wenjing Zhang, Zhijuan Lin, Fuyan Shi, Yujia Kong, Yanfeng Ren, Juncheng Lyu, Hao Qin, Hongqing Liu

**Affiliations:** 1 Department of Health Statistics, Key Laboratory of Medicine and Health of Shandong Province, School of Public Health, Weifang Medical University, Weifang 261053, China; 2 Key Laboratory for Immunology in Universities of Shandong Province, School of Basical Medicine, Weifang Medical University, Weifang 261053, China; 3 Weifang Key Laboratory for Food Nutrition and Safety, School of Public Health, Weifang Medical University, Weifang 261053, China

**Keywords:** *FASN* mutations, immunotherapies, melanoma, NSCLC, efficacy indicator

## Abstract

**Simple Summary:**

A key point in immunotherapies is to determine tumor cases that are sensitive to immune checkpoint inhibitors (ICI). Effective indicators could accurately evaluate the ICI treatment efficacy. Fatty acid synthase (FASN) is frequently mutated in tumor genomes. In this work, we consolidated genomic and clinical ICI data of melanoma and non-small cell lung cancer (NSCLC) samples, noticed that *FASN* mutations were linked to an elevated antitumor immunity, and were predictive of an improved ICI prognosis outcome and immunotherapeutic response rate. Our work offers a possible clinical indicator for assessing cancer ICI efficacy and selecting patients to receive immunotherapies.

**Abstract:**

Fatty acid synthase (FASN) acts as the central member in fatty acid synthesis and metabolism processes, which regulate oncogenic signals and tumor immunogenicity. To date, no studies have reported the connection of *FASN* mutations with ICI efficacy. In this study, from 631 melanoma and 109 NSCLC patients who received ICI treatments, we retrospectively curated multiomics profiles and ICI treatment data. We also explored the potential molecular biological mechanisms behind *FASN* alterations. In melanoma patients, *FASN* mutations were observed to associate with a preferable immunotherapeutic prognosis and response rate (both *p* < 0.01). These connections were further corroborated by the NSCLC patients (both *p* < 0.01). Further analyses showed that a favorable tumor immunogenicity and immune microenvironment were involved in *FASN* mutations. This work confirms the clinical immunotherapy implications of *FASN* mutation-mediated fatty acid metabolism and provides a possible indicator for immunotherapy prognosis prediction.

## 1. Introduction

Fatty acid (FA) synthesis and metabolism are important biological processes of cancer cells. Fatty acid biosynthesis or extracellular acquisition can not only promote tumor cell proliferation but also offer the energy source in the course of metabolic stress [1]. A common hallmark of tumor cells is FA metabolism reprogramming, which mainly comprises changes in de novo FA synthesis, FA transportation, and β-oxidation processes [2].

FA metabolism can be regulated by multiple oncogenic signaling to enhance tumor activity. A well-known dysregulated signaling in tumor is the PI3K–AKT axis, which associates with an elevated HER2 activity [3]. In HER2-positive tumors, HER2 upregulation produces a fatty acid synthase (FASN) activation subtype, which provides sustained FA synthesis and facilitates a tumor proliferative rate [4,5]. The activation of AKT can directly or indirectly modulate the genes associated with NADPH synthesis, thus conducing to lipogenesis [6,7]. Besides, PI3K–AKT signaling activates mTORC1 and mTORC2 [8,9], which are two regulators for promoting the expression of FA synthesis enzymes (e.g., FASN, ACC1, and ACLY) [10,11].

Previous studies demonstrated that FA biological processes are implicated with the tumor microenvironment, immune response, and immunotherapy efficacy. Enhanced lipid content in several myeloid immune cells (e.g., MDSC, DC, and TAM) has been reported to transform the immune response capacity of these cells to immunosuppressive function, thus mediating an immune-escape phenotype [12,13,14]. An elevatory FA accumulation in CD8^+^ T cells was connected to an enhanced expression of immune checkpoint PD-1, which has the ability to recognize tumor cells and predict favorable anti-PD-1 treatment outcomes [15]. SREBP-dependent lipid synthesis promotes functional specialization of regulatory T cells by targeting PD-1, which facilitates tumor proliferation and reduces immunotherapy response [16].

Fatty acid synthase (FASN) is a central member in FA metabolism and ultimately catalyzes seven malonyl-CoA and one acetyl-CoA molecules into FA16:0 (a main product of FA de novo synthesis) [17]. Taking into account its multifaceted roles in FA metabolism and oncogenic processes, multiple FASN inhibitions were developed for cancer treatment, such as first-generation [18,19] and next-generation agents [20,21]. These FASN-targeted agents were confirmed to reduce tumor proliferation and induce cell cycle arrest in breast and colorectal cancers. The combinatorial roles of the FASN inhibitor and conventional chemotherapy in breast cancer and astrocytoma are being evaluated under clinical trials [22].

To date, the roles of *FASN* alterations in cancer immune checkpoint inhibitor (ICI) treatment have never been reported. Considering that ICI therapies are broadly applied in melanoma and non-small cell lung cancer (NSCLC), we retrospectively integrated pre-treatment multiomics data and immunotherapy responses from melanoma and NSCLC patients to elucidate the clinical implications of *FASN* mutation-mediated FA metabolism reprogramming in cancer immunotherapy.

## 2. Materials and Methods

### 2.1. Collection of Samples

Pretreatment somatic mutational data, clinicopathologic features, and immune checkpoint inhibitor (ICI) treatment follow-up information for 631 melanoma [23,24,25,26,27,28,29,30] and 109 NSCLC samples [31,32] were retrospectively integrated (Tables S1 and S2). All included patients were treated with anti-CTLA-4, anti-PD-1/PD-L1, or combination agents. Pretreatment somatic mutation data were uniformly annotated with Oncotator [33]. Melanoma and NSCLC patients from the Cancer Genome Atlas (TCGA) were also obtained for corroboration. The transcriptomic expression data were used for elucidating possible biological mechanisms of *FASN* mutations.

### 2.2. Evaluation of Tumor Infiltration Immunocytes

To elaborate the different immunocyte abundance between *FASN* mutated and wild-type subgroups, we used two methods to calculate a detailed infiltration proportion of lymphocyte subtypes. CIBERSORT was proposed to evaluate the infiltration proportion of 22 immunocyte types with LM22 signature, which contains 547 characteristic genes [34]. Angelova et al.’s method applied an 812-feature-gene panel to calculate 31 distinct immunocytes’ infiltrating levels [35]. Specific feature genes in Angelova et al.’s method are illustrated in Table S3.

### 2.3. Tumor Microenvironment-Based Signatures

Previous studies have reported that multiple immune and immunocyte-related signatures play vital roles in cancer immunogenicity and progression. We therefore collected several representative signatures in Table S4.

### 2.4. GSVA and GSEA

Single sample gene set enrichment analysis (ssGSEA), which is one type of gene set variation analysis was used to estimate the enrichment scores of curated immune and lymphocyte-related signatures under the R GSVA package [36]. Whole-genome expression differential analysis of *FASN* mutated versus wild-type subgroups was conducted with the R DESeq2 package [37]. The obtained *t* values were subsequently regarded the inputs to perform GSEA analysis to obtain dysregulated signaling pathways of patients with *FASN* mutations. Background pathways were acquired from the Hallmark database and downloaded from the Molecular Signatures Database (MSigDB) [38].

### 2.5. Statistical Analysis

The R software was applied to perform relevant calculations and achieve plots. Mutational signatures were extracted by using the method proposed by Kim et al. [39]. A waterfall plot was used to exhibit specific gene mutational patterns and implemented by the maftools R package [40]. A heatmap representation of distinct immune signatures in *FASN* mutant and wild-type groups was completed with the pheatmap package. The Wilcoxon rank-sum test (Wilcoxon test) and Fisher exact test were applied to respectively evaluate the association of continuous and categorical variables with *FASN* mutations.

## 3. Results

### 3.1. FASN Mutational Status in Melanoma

**Figure 1:**
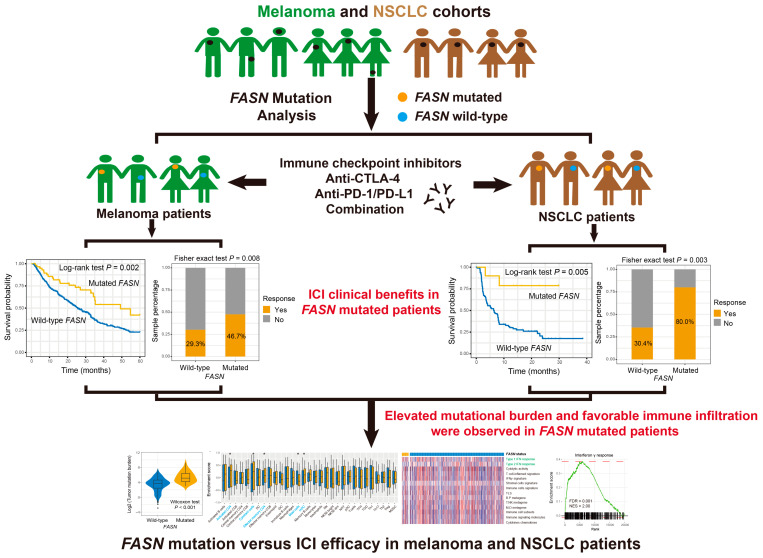
Flowchart of this study. Association of *FASN* mutations with ICI efficacy in melanoma and NSCLC.

A flowchart for this work is exhibited in Figure 1. Of the pooled 631 melanoma patients, 193 (30.6%) were ICI responders. The main substitution pattern for these melanoma patients was C > T (Figure S1). Detailed mutation patterns of melanoma driver genes in relation to *FASN* mutations are presented in Figure S1. We noticed that *FASN* is mutant in 60 of 631 melanoma samples (9.5%). Amino acid change information of *FASN* alterations is shown in Figure S2.

### 3.2. FASN Mutations Predict Favorable ICI Efficacy in Melanoma

**Figure 2:**
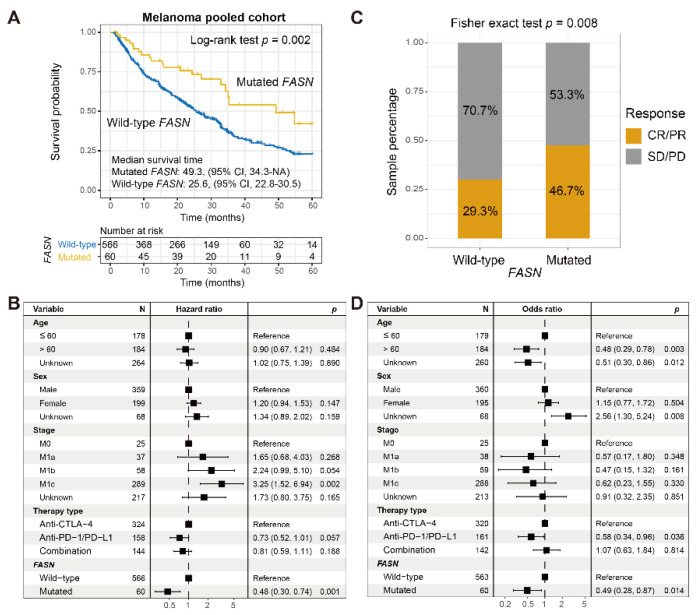
*FASN* mutations predictive of ICI outcome and response in melanoma. (**A**) Kaplan–Meier survival curves stratified based on *FASN* mutational status. (**B**) Multivariate Cox regression model of *FASN* mutations was conducted with age, sex, stage, and therapy type taken into account. (**C**) Distinct ICI response rates of *FASN* mutated versus wild-type subgroups. (**D**) Multivariate logistic regression model of *FASN* mutations was conducted with clinical factors taken into account.

*FASN* mutated melanoma patients exhibited a preferable immunotherapeutic survival than the rest of the patients (*p* = 0.002; Figure 2A). This connection was still significant in a multivariable adjusted Cox regression (*p* = 0.001; Figure 2B). Survival predictive capacities for *FASN* alterations in single ICI cohorts (Figure S3) and specific therapies (Figure S4) were separately shown. Patients with *FASN* mutations also exhibited an immunotherapeutic response advantage over the rest of the patients (response rate: 46.7% vs. 29.3%, *p* = 0.008; Figure 2C). A multivariable adjusted analysis further confirmed this connection (*p* = 0.014; Figure 2D).

### 3.3. FASN Mutations Predict Melanoma Elevated Mutational Burden

**Figure 3:**
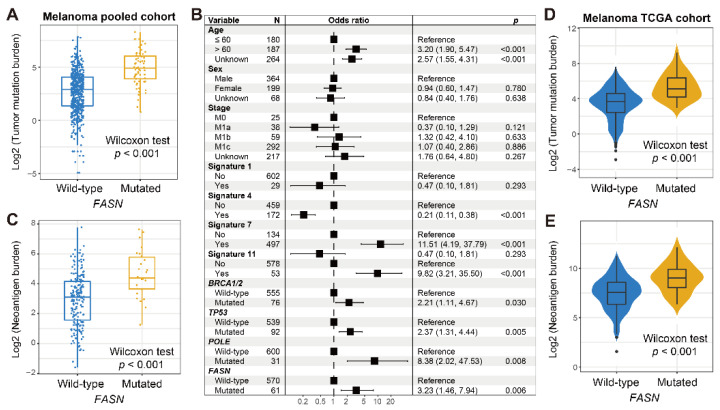
Association between *FASN* mutations and mutational burden in melanoma. (**A**) Distinct distribution of TMB in *FASN* two subgroups in the pooled cohort. (**B**) Multivariate logistic regression model of *FASN* mutations was conducted with clinical factors, extracted mutational signatures, and genomic maintenance regulator mutations taken into account. (**C**) Distinct distribution of NB in *FASN* two subgroups. Association of *FASN* mutations with (**D**) TMB and (**E**) NB in the TCGA cohort.

A higher tumor mutation burden (TMB) was revealed to involve in the favorable ICI effect. We therefore explored the connection of *FASN* mutations with TMB. As shown in Figure 3A, a significantly higher TMB was observed in *FASN* mutated samples (*p* < 0.001). Genomic stability is largely influenced by mutational signatures. We thus extracted four potential signatures from melanoma mutational profiles by using the NMF algorithm. Detailed mutational activities of each signature for each sample are presented in Table S5. To obtain a real association of *FASN* mutations with TMB, we conducted a multivariable adjusted analysis with clinicopathologic characteristics, identified mutational signatures, and alterations in DNA damage regulators taken into consideration. Results showed the elevated TMB still existed in *FASN* mutated patients (*p* = 0.006; Figure 3B). Moreover, *FASN* alterations were connected with a higher neoantigen burden (NB) (*p* < 0.001; Figure 3C). Consistently, in the TCGA melanoma cohort, *FASN* mutated patients exhibited both increased TMB and NB (both *p* < 0.001; Figure 3D,E).

### 3.4. Corroboration in NSCLC

**Figure 4:**
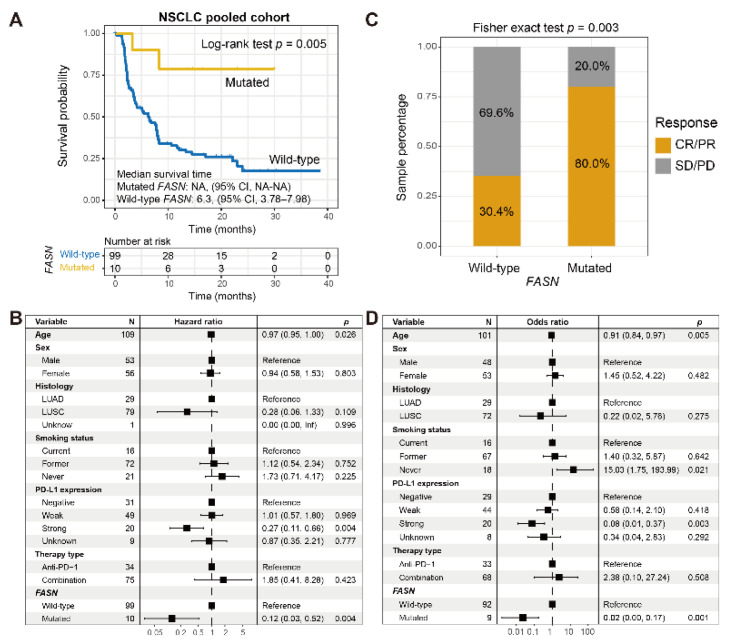
*FASN* mutations predictive of ICI outcome and response in NSCLC. (**A**) Kaplan–Meier survival curves stratified based on *FASN* mutational status. (**B**) Multivariate Cox regression model of *FASN* mutations was conducted with multiple confounding factors taken into account. (**C**) Distinct ICI response rates of *FASN* mutated versus wild-type subgroups. (**D**) Multivariate logistic regression model of *FASN* mutations was conducted with multiple confounding factors taken into account.

Of the 109 curated NSCLC samples, 36 (33.0%) exhibited the ICI responsive status. Ten (9.2%) of 109 patients harbored *FASN* mutations. Univariate survival analysis revealed that *FASN* mutated NSCLC samples exhibited an improved immunotherapeutic survival than other patients (*p* = 0.005; Figure 4A). A consistent result was obtained in a multivariable adjusted Cox analysis with confounding factors taken into account (*p* = 0.004; Figure 4B). Associations between *FASN* mutations and ICI outcomes in single cohorts (Figure S5) were presented. Further exploration suggested that an immunotherapeutic response advantage was also noticed in *FASN* mutated groups (80.0% vs. 30.4%, *p* = 0.003; Figure 4C). Additionally, this association remained more significant after controlling for multiple variables (*p* = 0.001; Figure 4D).

**Figure 5:**
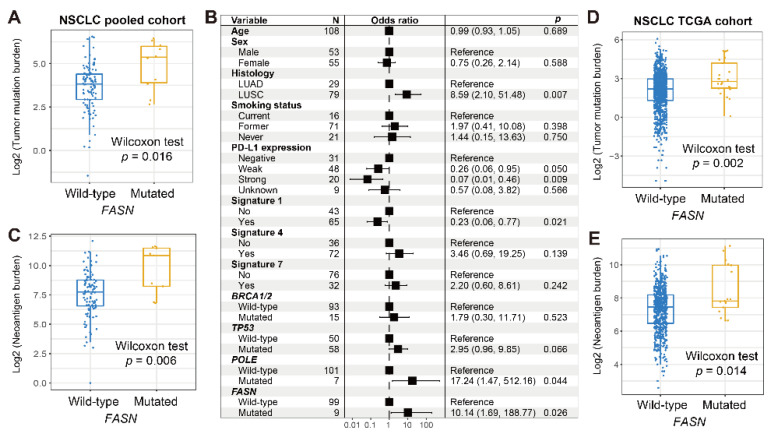
Association between *FASN* mutations and mutational burden in NSCLC. (**A**) Distinct distribution of TMB in *FASN* two subgroups in the pooled cohort. (**B**) Multivariate logistic regression model of *FASN* mutations was conducted with clinical factors, extracted mutational signatures, and genomic maintenance regulator mutations taken into account. (**C**) Distinct distribution of NB in *FASN* two subgroups. Association of *FASN* mutations with (**D**) TMB and (**E**) NB in the TCGA cohort.

An enhanced TMB was detected in the *FASN* mutant group (*p* = 0.016; Figure 5A). Three mutational signatures were determined against NSCLC mutation data (Table S6). A multivariable logistic analysis with clinicopathologic factors, mutational signatures, and DNA repair regulator mutations was incorporated; the link between *FASN* alterations and elevated TMB was still noticed (*p* = 0.026; Figure 5B). *FASN* mutations were also connected with an increased NB (*p* = 0.006; Figure 5C). Similarly, based on mutation data from the TCGA NSCLC cohort, the significantly enhanced TMB and NB were also found in the *FASN* mutant subgroup (*p* = 0.002 and 0.014; Figure 5D,E).

### 3.5. Immunological Features and Pathway Enrichment behind FASN Mutations

**Figure 6:**
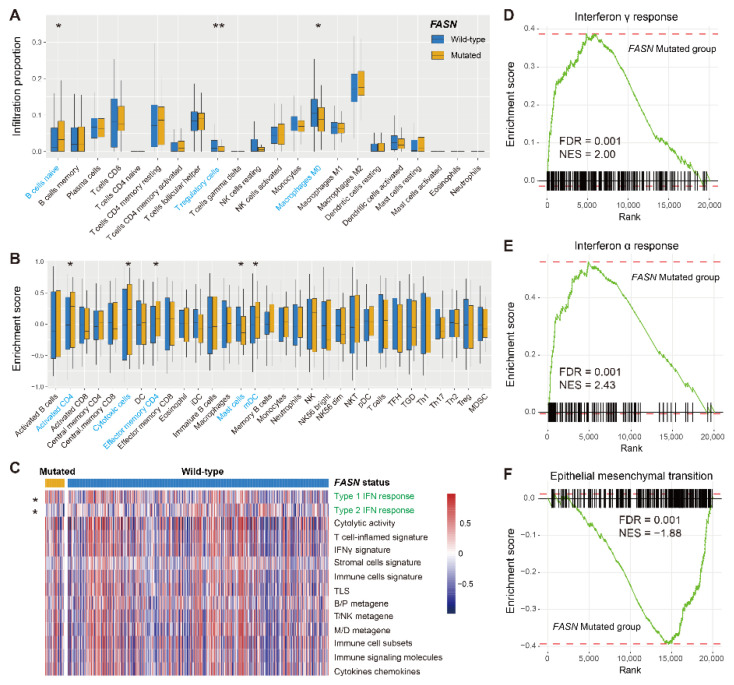
Immune infiltration and pathway enrichment associated with *FASN* mutations in melanoma. (**A**) Distinct infiltration abundance of 22 immunocytes of *FASN* mutated and wild-type groups evaluated with the CIBERSORT algorithm. Immunocytes highlighted with blue are significantly differentially infiltrated. (**B**) Distinct infiltration abundance of 31 immunocytes of *FASN* mutated and wild-type groups evaluated with Angelova et al.’s method. (**C**) Heatmap representation of distinct enrichment scores of 14 collected immune-related signatures in *FASN* two subgroups. Signatures highlighted with green are significantly differentially enriched. (**D**–**F**) Significantly enriched signaling pathways associated with *FASN* mutations. * *p* < 0.05, ** *p* < 0.01.

To further understand the possible immunological mechanisms behind *FASN* mutations in melanoma, we performed a multiangle immunology and pathway analysis. Based on the results from the CIBERSORT algorithm (Figure 6A), we observed a significantly enhanced infiltration of naive B cells and a decreased infiltration of T regulatory cells in *FASN* mutated melanoma patients (both *p* < 0.05). Besides, *FASN* mutations were also associated with a decreased M0 macrophage infiltration (*p* < 0.05). Similarly, under Angelova et al.’s approach (Figure 6B), *FASN* mutated patients exhibited the elevated infiltration abundance of prinflammatory immunocytes (e.g., activated CD4 T cells, cytotoxic cells, effector memory CD4 T cells, and mDC cells) and decreased the infiltration of immunosuppressive mast cells (all *p* < 0.05).

A heatmap was subsequently achieved to illustrate the different enrichment scores of immune-relevant signatures in *FASN* two subgroups (Figure 6C). Results showed that both types I and II interferon responses were significantly enriched in the *FASN* mutated group (both *p* < 0.05). GSEA further confirmed these findings that interferon γ and α responses (which belong to interferons II and I response) were enriched in the *FASN* mutant subgroup (both NES > 2, FDR = 0.001; Figure 6D,E and Figure S6). The immunosuppressive signaling pathway of epithelial–mesenchymal transition (EMT) was absent in the *FASN* mutated subgroup (NES = −1.88, FDR = 0.001; Figure 6F and Figure S6).

We finally conducted immunocyte infiltrating and GSEA analyses for NSCLC samples. Consistent with the findings obtained from melanoma, a favorable infiltration of immune response lymphocytes (e.g., activated CD4/CD8 T cells, resting NK cells, and M1 macrophages) (all *p* < 0.05; Figure S7A,B), an elevated enrichment of interferon γ/α responses (Figure S7C), and a decreased enrichment of immune-inhibited pathways (e.g., TGFβ and EMT signals) (Figure S7C) were noticed in *FASN* mutated NSCLC samples.

## 4. Discussion

FA synthesis and metabolism play vital roles in cancer immunogenicity and immunotherapy, since FASN is a central member of FA metabolism processes, and no studies have reported the connection between *FASN* alterations and ICI therapy efficacy. Therefore, we integrated multiomics and immunotherapy data from melanoma and NSCLC samples, and found that *FASN* mutations could predict a preferable immunotherapeutic survival and response, which proposes a clinically potential indicator for choosing tumor patients to treat with immunotherapies.

The classical role of FASN is to control FA de novo synthesis and metabolism. Recent multiple studies have revealed its immune regulation roles in cancers. A study reported that FASN-mediated FA synthesis promotes functional maturation of T regulatory cells, and thus induces immune response suppression and accelerated tumor proliferation [16]. Consistently, in ovarian cancer, FASN suppressed immune activity via decreasing the ability of dendritic cells to sustain T lymphocytes [41]. Indeed, *FASN* per se was identified as a poor prognosticator and associated with an inferior immune infiltration in gastric cancer [42]. A FASN-targeted inhibitor, C75, boosted antitumor immune capacity and blockaded tumor proliferation under the setting with the PI3Kα inhibitor CYH33 in breast cancer [43]. Similarly, the enhanced immune response ability of another FASN inhibitor, orlistat, was also revealed in melanoma [44]. In an analysis conducted by Zhang et al., an immune-related signature contained *FASN* expression was determined to be associated with bladder cancer survival and immunotherapy response [45]. Collectively, FASN plays an immunosuppressive role in tumors, and inhibition of FASN by chemotherapy agents could transform the suppressive status to an immune-activated environment. Hypothetically, mutations in *FASN* may change or inactivate the functions of FASN, which results in a favorable immune microenvironment and immunotherapy efficacy obtained from our study.

In this study, *FASN* mutations were linked to the preferable immunotherapeutic survival in both tumors under an ICI treatment setting. We also analyzed the association of *FASN* mutations with treatment outcomes in both tumors receiving conventional chemotherapies in the TCGA cohort. However, no significant survival differences between *FASN* two subgroups were found (both *p* > 0.05; Figure S8). The above results demonstrate that *FASN* mutations might predict the better therapeutic response in the setting of immunotherapies. Clinical significances of *FASN* alterations in other therapies are needed for validation.

A higher TMB was demonstrated to be linked to a preferable immunotherapeutic outcome in multiple cancers [46,47,48,49]. However, accurate evaluation of TMB needs to perform tumor whole-exome sequencing, and cut-off values for TMB to stratify patients into high and low subgroups vary in distinct cancers [50]. Multiple studies have revealed that mutations in single genes, such as *POLE* [51], *TP53* [52], *FAT1* [53], *MUC16* [54], and *PBRM1* [55], may predict the elevated TMB and ICI efficacy. Our study discovered that *FASN* mutations were connected with a significantly enhanced mutational burden and a better ICI efficacy, which provides a potential marker for clinical immunotherapies.

A recent study reported that overexpression of FASN was linked to a worse survival outcome in patients with colorectal cancer [56], which indicates that FASN-mediated fatty acid synthase and metabolism play a negative role and contribute to the poor prognosis of cancers. Lyu et al. demonstrated that high mutations in fatty acid metabolism induced a favorable outcome in small-cell lung cancer [57]; this may be attributed to the fatty acid metabolism mutation-induced function inactivation.

We also explored the association between *FASN* mutations and *FASN* expression in melanoma and NSCLC patients under the TCGA cohort to elucidate how *FASN* mutation alters its transcriptional expression. We observed that melanoma patients with *FASN* mutations exhibited a significantly decreased *FASN* expression (Wilcoxon rank-sum test, *p* = 0.006; Figure S9A), which is consistent with the evidence that patients with low *FASN* expression responded better to ICI treatments. However, in NSCLC, no significant association between *FASN* mutations and *FASN* expression was observed (Wilcoxon rank-sum test, *p* = 0.071; Figure S9B). Further exploration and functional validation are necessary.

Several limitations are found in this work. First, the mutation data and immunotherapy information of both melanoma and NSCLC patients were retrospectively collected from multiple datasets, which might introduce some deviation. Second, association of *FASN* mutations with ICI efficacy was discovered and confirmed only in two cancer types, and no additional cancers are available. Three, in-depth functional experiments are warranted.

## 5. Conclusions

Overall, we integrated genomic and clinical data from melanoma and NSCLC patients and found that *FASN* mutations might predict a preferable immunotherapeutic efficacy. Findings gleaned from our study suggest that *FASN* mutation-mediated FA metabolism may be considered as an indicator for cancer immunotherapy efficacy evaluation.

## Data Availability

All datasets employed in this work were publicly acquired.

## Supplementary Materials

The following supporting information can be downloaded at: https://www.mdpi.com/article/10.3390/cancers14225638/s1: Figure S1: Waterfall plot representation of mutation information of *FASN* and specific genes in melanoma; Figure S2: Amino acid changes associated with *FASN* alterations in melanoma patients; Figure S3: Survival predictive capacities of *FASN* mutations in single melanoma datasets; Figure S4: Survival predictive capacities of *FASN* mutations in different melanoma ICI treatments; Figure S5: Survival predictive capacities of *FASN* mutations in different NSCLC ICI treatments; Figure S6: Pathway analysis results of *FASN* two subgroups based on the GSEA in melanoma; Figure S7: Immunological features and pathway enrichment behind *FASN* alterations in NSCLC. (A) CIBERSORT algorithm infers distinct infiltrating levels of 22 immune cells in *FASN* two subgroups. (B) Angelova et al.’s method infers distinct infiltrating levels of 31 immune cells in *FASN* two subgroups. (C) Pathway analysis results of *FASN* two subgroups based on the GSEA; Figure S8: Survival predictive capacities of *FASN* mutations in (A) melanoma and (B) NSCLC samples obtained from the TCGA cohort; Figure S9: Association of *FASN* mutations with *FASN* expression in (A) melanoma and (B) NSCLC patients based on the TCGA cohort; Table S1: Clinicopathologic features of 631 included melanoma samples; Table S2: Clinicopathologic features of 109 included NSCLC samples; Table S3: Representative genes for distinct immunocyte subtypes; Table S4: Representative genes for distinct immunogenicity-relevant signatures; Table S5: The determined four mutational signatures and corresponding mutational activities in melanoma; Table S6: The determined three mutational signatures and corresponding mutational activities in NSCLC.
