# Supplementary material for: Fatty Acid Synthase Mutations Predict Favorable Immune Checkpoint Inhibitor Outcome and Response in Melanoma and Non-Small Cell Lung Cancer Patients
Source: Cancers (Basel). 2022 Nov 17;14(22):5638. doi: 10.3390/cancers14225638 (PMC9688165; doi:10.3390/cancers14225638)
Supplement: Supplementary file 1 [file cancers-14-05638-s001.zip › Supplementary Figures.pdf]

Supplementary Figures

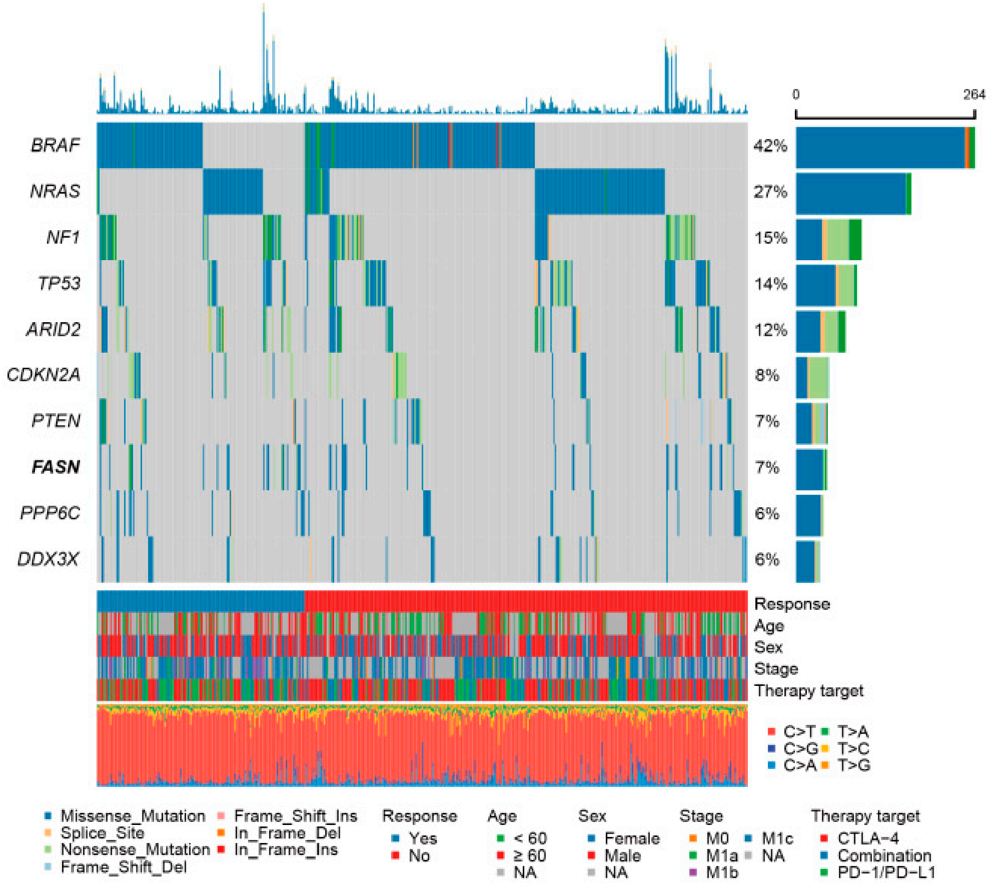

Figure S1. Waterfall plot representation of mutation information of *FASN* and specific genes in melanoma.

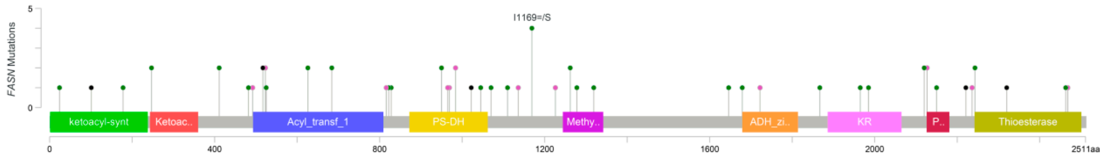

Figure S2. Amino acid changes associated with *FASN* alterations in melanoma patients.

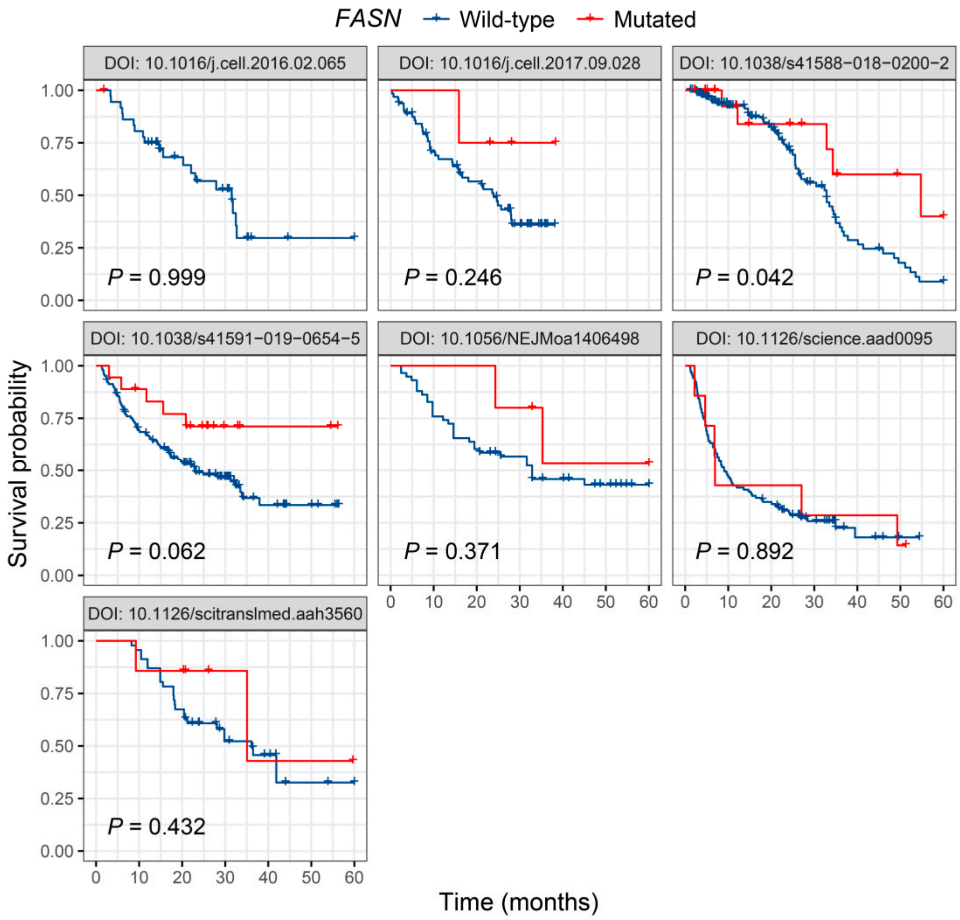

Figure S3. Survival predictive capacities of *FASN* mutations in single melanoma datasets.

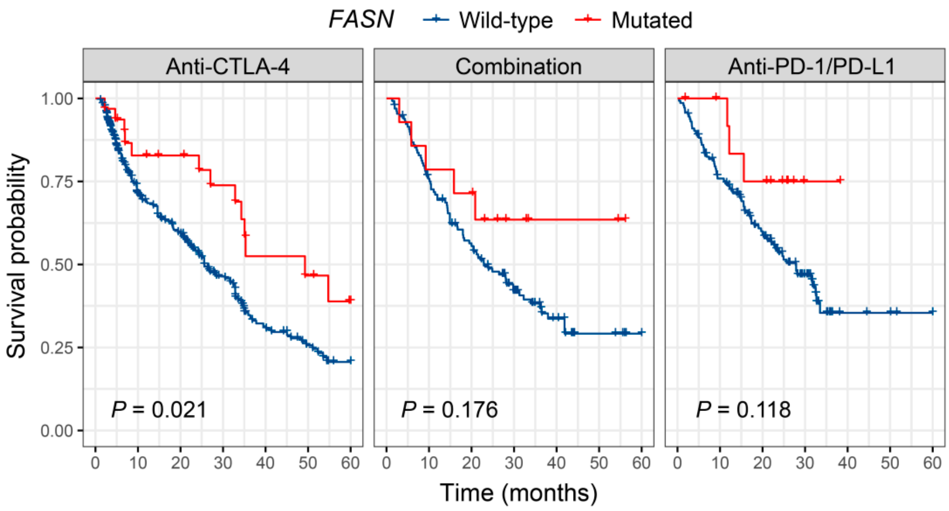

Figure S4. Survival predictive capacities of *FASN* mutations in different melanoma ICI treatments.

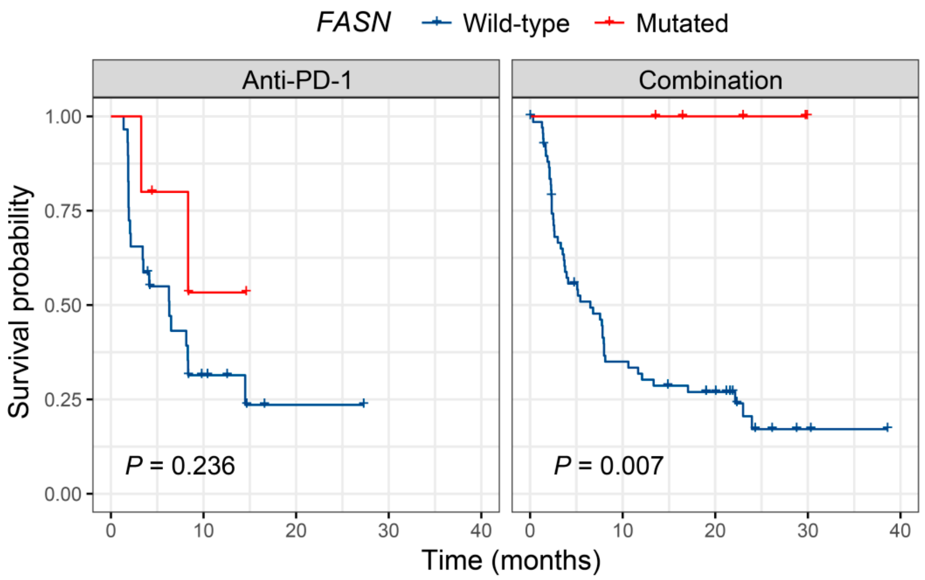

Figure S5. Survival predictive capacities of *FASN* mutations in different NSCLC ICI treatments.

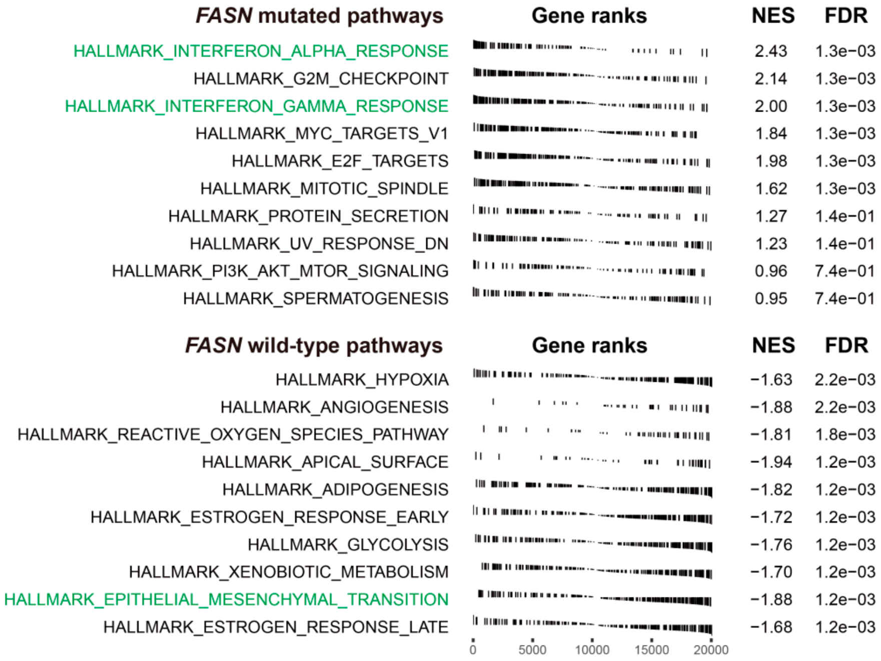

Figure S6. Pathway analysis results of *FASN* two subgroups based on the GSEA in melanoma.

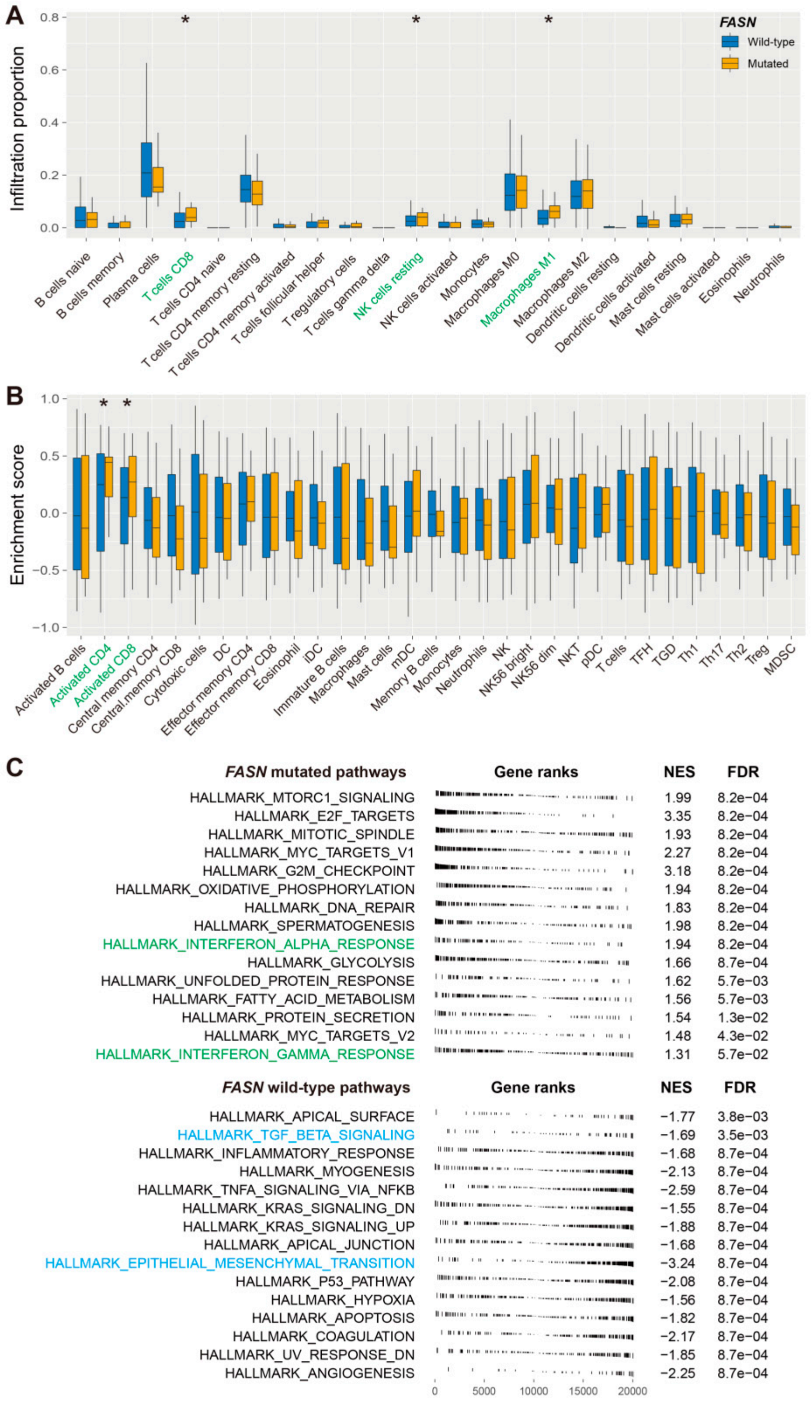

Figure S7. Immunological features and pathway enrichment behind *FASN* alterations in NSCLC. (A) CIBERSORT algorithm infers distinct infiltrating levels of 22 immune cells in *FASN* two subgroups. (B) Angelova *et al.* method infers distinct infiltrating levels of 31 immune cells in *FASN* two subgroups. (C) Pathway analysis results of *FASN* two subgroups based on the GSEA.

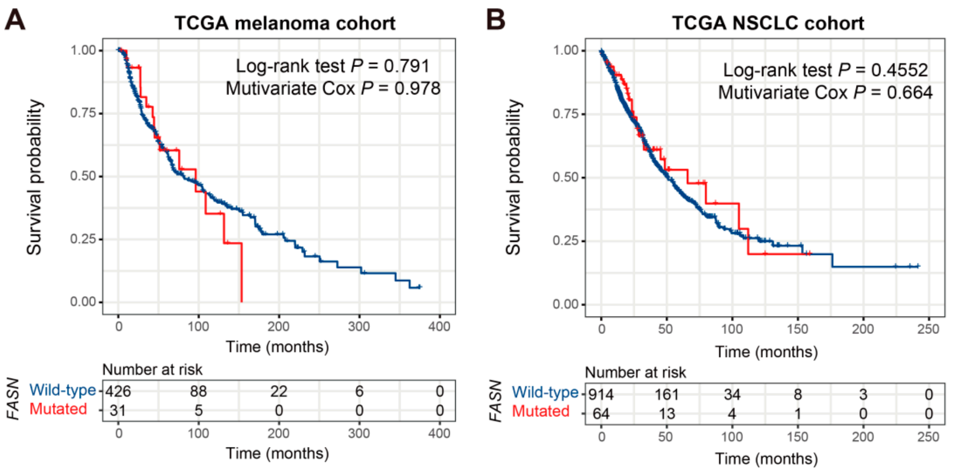

Figure S8. Survival predictive capacities of *FASN* mutations in (A) melanoma and (B) NSCLC samples obtained from the TCGA cohort.

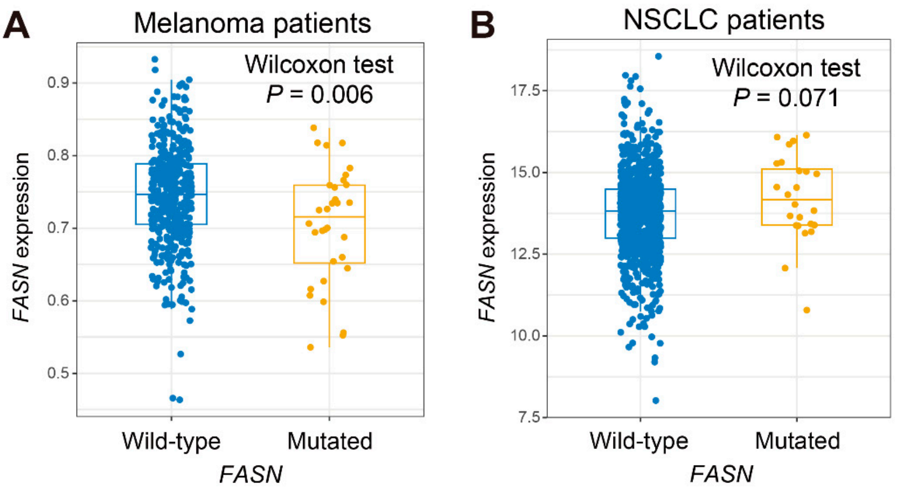

Figure S9. Association of *FASN* mutations with *FASN* expression in (A) melanoma and (B) NSCLC patients based on the TCGA cohort.
